# Di­aqua­bis­(4-ferrocenyl-1,1,1-tri­fluoro-4-oxobutan-2-olato)cobalt(II)

**DOI:** 10.1107/S2414314620012407

**Published:** 2020-09-11

**Authors:** Shujing Wang, Heguo Han, Yuyang Han

**Affiliations:** a Department of Chemistry, Anhui University, Hefei, Anhui 230039, People’s Republic of China; University of Aberdeen, Scotland

**Keywords:** crystal structure, cobalt(II) complex, tri­fluoro­methyl-β-diketone ferrocene ligand

## Abstract

In the title compound, [CoFe_2_(C_5_H_5_)_2_(C_9_H_5_F_3_O_2_)_2_(H_2_O)_2_], the central Co atom has an octa­hedral coordination geometry defined by two chelating tri­fluoro­methyl-β-diketone ferrocene ligands and two aqua ligands, with the latter in a *cis* disposition. The Cp rings in both ferrocene groupings are close to eclipsed. In the crystal, O—H⋯O hydrogen bonds link the mol­ecules into [100] chains.

## Abstract 2

In the title compound, the coordinated water mol­ecules are in a *cis* disposition and the Cp rings of the ferrocene groups are close to eclipsed.

## Graphical abstract

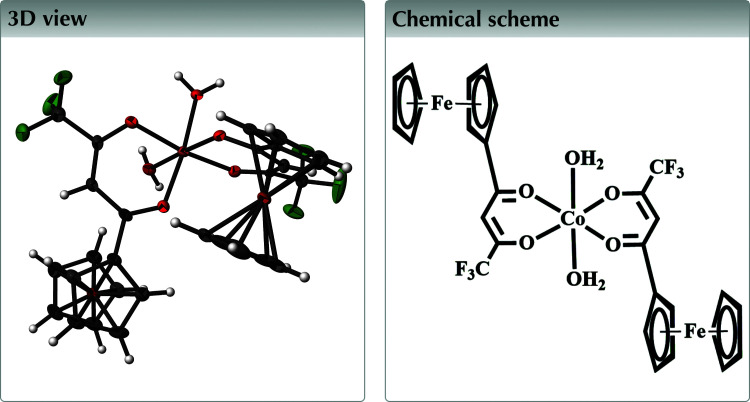

## Structure description

β-Diketones are good chelating ligands for transition-metal ions and rare-earth metal ions and can possess pendant groups such as ferrocene. The resulting complexes may have applications in the fields of electroluminescent devices, environmental sensors, photodynamic therapy and biological imaging (*e.g.*, Zheng *et al.*, 2019 ▸). As part of our work in this area, we now describe the synthesis and structure of the title cobalt(II) complex.

**Figure 1:**
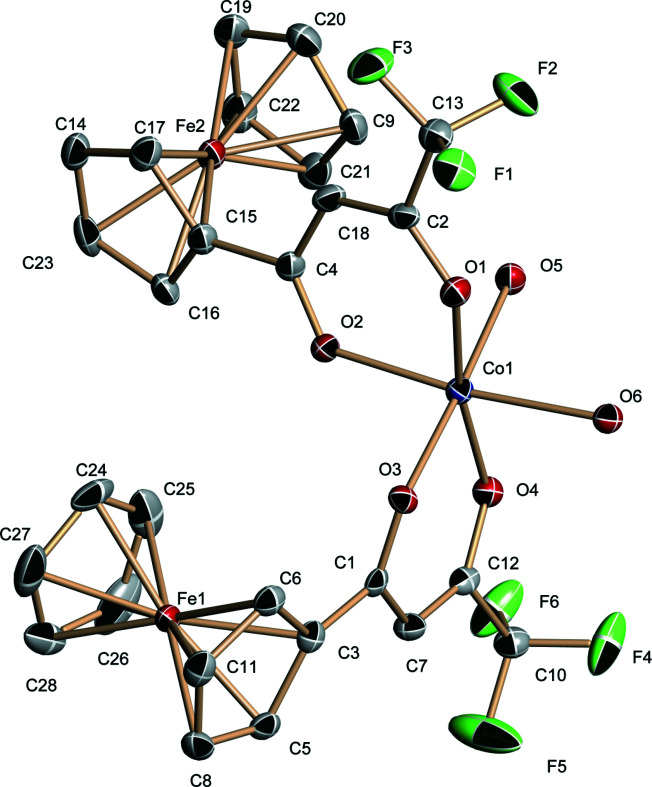
The mol­ecular structure of the title compound with displacement ellipsoids drawn at the 30% probability level; H atoms are omitted for clarity.

The cobalt(II) atom has a octa­hedral coordination environment defined by two tri­fluoro­methyl-β-diketone ferrocene ligands and two water ligands (Fig. 1 ▸) with the water mol­ecules in a *cis* disposition: the dihedral angle between the carbon-atom skeletons of the diketone ligands is 81.73 (12)°. The Cp rings of both ferrocene groups are in nearly eclipsed conformations.

**Figure 2:**
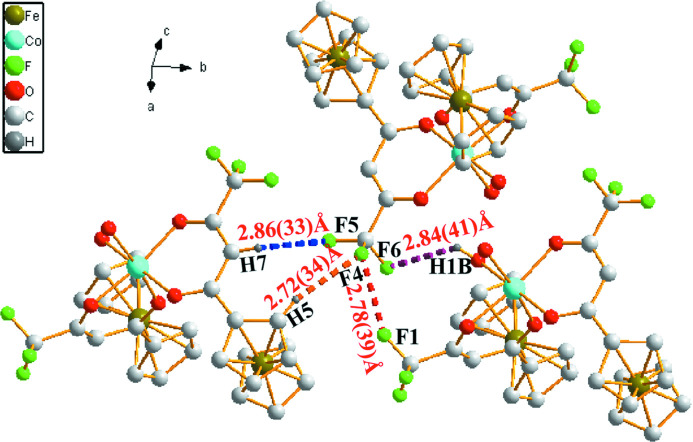
Partial packing diagram of the title compound showing significant H⋯F and F⋯F contacts involving the C10/F4/F5/F6 group as dashed lines.

In the crystal, the mol­ecules are linked into [100] chains by O—H⋯O hydrogen bonds arising from the water mol­ecules (Table 1 ▸, Fig. 2 ▸); an intra­molecular C—H⋯O and an inter­molecular C—H⋯F link are also present. A short inter­molecular F2⋯F6(1 − *x*, 1 − *y*, 1 − *z*) contact [2.783 (4) Å; van der Waals radius sum = 2.94 Å].

## Synthesis and crystallization

In a 250 ml round-bottom flask, tri­fluoro­methyl-β-diketone ferrocene (0.52 g 1.6 mmol), tri­ethyl­amie (0.25 g 2.45 mmol) and cobalt acetate (0.13 g 0.5 mmol) were dissolved in 100 ml of methanol and the mixture was stirred at 343 K for 12 h and then cooled to room temperature. A red solid was obtained by suction filtration. Crystals for X-ray analysis were obtained by recrystallization from methanol solution.

## Refinement

Crystal data, data collection and structure refinement details are summarized in Table 2 ▸.

## Supplementary Material

Crystal structure: contains datablock(s) I. DOI: 10.1107/S2414314620012407/hb4360sup1.cif

Structure factors: contains datablock(s) I. DOI: 10.1107/S2414314620012407/hb4360Isup3.hkl

CCDC reference: 2031062

Additional supporting information:  crystallographic information; 3D view; checkCIF report

## Figures and Tables

**Table 1:** Hydrogen-bond geometry (Å, °)

*D*—H⋯*A*	*D*—H	H⋯*A*	*D*⋯*A*	*D*—H⋯*A*
O5—H5*A*⋯O6^i^	0.89 (4)	1.97 (4)	2.797 (5)	154 (6)
O5—H5*B*⋯O4^i^	0.88 (4)	2.01 (4)	2.781 (4)	146 (5)
O6—H6*A*⋯O3^ii^	0.88 (4)	1.91 (5)	2.739 (5)	156 (4)
O6—H6*B*⋯O1^ii^	0.89 (6)	1.99 (6)	2.741 (5)	141 (5)
C25—H25⋯O2	0.93	2.57	3.467 (7)	163
C28—H28⋯F3^iii^	0.93	2.55	3.449 (8)	163

Symmetry codes: (i) 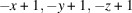 ; (ii) 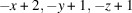 ; (iii) 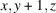 .

**Table 2:** Experimental details

Crystal data	
Chemical formula	[CoFe_2_(C_5_H_5_)_2_(C_9_H_5_F_3_O_2_)_2_(H_2_O)_2_]
*M*_r_	741.10
Crystal system, space group	Triclinic, *P* 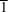
Temperature (K)	298
*a*, *b*, *c* (Å)	7.725 (3), 12.721 (3), 14.609 (4)
α, β, γ (°)	70.67 (3), 82.88 (3), 80.46 (3)
*V* (Å^3^)	1332.3 (8)
*Z*	2
Radiation type	Cu *K*α
μ (mm^−1^)	14.22
Crystal size (mm)	0.30 × 0.20 × 0.10

Data collection	
Diffractometer	Stoe Stadivari
Absorption correction	Multi-scan (*X-AREA*; Stoe & Cie, 2018 ▸)
*T*_min_, *T*_max_	0.791, 1
No. of measured, independent and observed [*I* > 2σ(*I*)] reflections	10376, 4786, 3741
*R*_int_	0.031
(sin θ/λ)_max_ (Å^−1^)	0.609

Refinement	
*R*[*F* ^2^ > 2σ(*F* ^2^)], *wR*(*F* ^2^), *S*	0.049, 0.127, 1.05
No. of reflections	4786
No. of parameters	404
No. of restraints	4
H-atom treatment	H atoms treated by a mixture of independent and constrained refinement
Δρ_max_, Δρ_min_ (e Å^−3^)	0.88, −0.43

Computer programs: *X-AREA* (Stoe & Cie, 2018 ▸), *olex2.solve* (Bourhis *et al.*, 2015 ▸), *SHELXL* (Sheldrick, 2015 ▸) and *OLEX2* (Dolomanov *et al.*, 2009 ▸).

## full crystallographic data

### Crystal data

**Table:**

[CoFe_2_(C_5_H_5_)_2_(C_9_H_5_F_3_O_2_)_2_(H_2_O)_2_]	*Z* = 2
*M**_r_* = 741.10	*F*(000) = 746
Triclinic, *P*1	*D*_x_ = 1.847 Mg m^−^^3^
*a* = 7.725 (3) Å	Cu *K*α radiation, λ = 1.54186 Å
*b* = 12.721 (3) Å	Cell parameters from 1567 reflections
*c* = 14.609 (4) Å	θ = 2.2–27.1°
α = 70.67 (3)°	µ = 14.22 mm^−^^1^
β = 82.88 (3)°	*T* = 298 K
γ = 80.46 (3)°	Block, black
*V* = 1332.3 (8) Å^3^	0.30 × 0.20 × 0.10 mm

### Data collection

**Table:**

Stoe Stadivari diffractometer	3741 reflections with *I* > 2σ(*I*)
Detector resolution: 5.81 pixels mm^-1^	*R*_int_ = 0.031
rotation method, ω scans	θ_max_ = 69.8°, θ_min_ = 3.2°
Absorption correction: multi-scan (*X-AREA*; Stoe & Cie, 2018)	*h* = −9→8
*T*_min_ = 0.791, *T*_max_ = 1	*k* = −15→12
10376 measured reflections	*l* = −14→17
4786 independent reflections	

### Refinement

**Table:**

Refinement on *F*^2^	Primary atom site location: iterative
Least-squares matrix: full	Hydrogen site location: mixed
*R*[*F*^2^ > 2σ(*F*^2^)] = 0.049	H atoms treated by a mixture of independent and constrained refinement
*wR*(*F*^2^) = 0.127	*w* = 1/[σ^2^(*F*_o_^2^) + (0.0684*P*)^2^ + 2.1404*P*] where *P* = (*F*_o_^2^ + 2*F*_c_^2^)/3
*S* = 1.05	(Δ/σ)_max_ < 0.001
4786 reflections	Δρ_max_ = 0.88 e Å^−^^3^
404 parameters	Δρ_min_ = −0.43 e Å^−^^3^
4 restraints	

### Special details

**Table:**

Geometry. All esds (except the esd in the dihedral angle between two l.s. planes) are estimated using the full covariance matrix. The cell esds are taken into account individually in the estimation of esds in distances, angles and torsion angles; correlations between esds in cell parameters are only used when they are defined by crystal symmetry. An approximate (isotropic) treatment of cell esds is used for estimating esds involving l.s. planes.
Refinement. The water H atoms were located in difference maps and their positions were freely refined. The C-bound H atoms were geometrically placed (C—H = 0.93 Å) and refined as riding atoms.

### Fractional atomic coordinates and isotropic or equivalent isotropic displacement parameters (Å^2^)

**Table:**

	*x*	*y*	*z*	*U*_iso_*/*U*_eq_	
Fe1	1.04875 (9)	0.97569 (6)	0.25193 (5)	0.01775 (18)	
Co1	0.75176 (9)	0.59847 (6)	0.40437 (5)	0.01579 (18)	
Fe2	0.41805 (9)	0.68982 (6)	0.06889 (5)	0.01960 (19)	
F1	1.1799 (4)	0.3465 (2)	0.2975 (2)	0.0325 (7)	
O4	0.5761 (4)	0.6874 (2)	0.4767 (2)	0.0187 (7)	
O3	0.9383 (4)	0.6985 (2)	0.4060 (2)	0.0169 (6)	
O6	0.8032 (4)	0.4822 (3)	0.5508 (2)	0.0187 (7)	
O2	0.7000 (4)	0.6910 (2)	0.2635 (2)	0.0184 (7)	
F3	1.0466 (4)	0.3623 (3)	0.1729 (2)	0.0385 (8)	
O1	0.9307 (4)	0.4897 (3)	0.3551 (2)	0.0194 (7)	
O5	0.5584 (4)	0.5023 (3)	0.3964 (2)	0.0198 (7)	
F2	0.9375 (4)	0.2795 (2)	0.3160 (2)	0.0413 (8)	
F6	0.3060 (4)	0.8234 (3)	0.5387 (2)	0.0476 (9)	
F4	0.4611 (4)	0.7265 (3)	0.6544 (2)	0.0484 (9)	
F5	0.4848 (5)	0.8988 (3)	0.5881 (3)	0.0724 (14)	
C1	0.9093 (6)	0.7927 (4)	0.4211 (3)	0.0163 (9)	
C2	0.9182 (6)	0.4767 (4)	0.2728 (3)	0.0183 (9)	
C3	1.0453 (6)	0.8669 (4)	0.3887 (3)	0.0172 (9)	
C4	0.7257 (6)	0.6514 (4)	0.1939 (3)	0.0191 (10)	
C5	1.0342 (6)	0.9798 (4)	0.3894 (3)	0.0202 (10)	
H5	0.941577	1.017422	0.418284	0.024*	
C6	1.2095 (6)	0.8433 (4)	0.3352 (3)	0.0198 (10)	
H6	1.251640	0.776175	0.322669	0.024*	
C7	0.7500 (6)	0.8293 (4)	0.4712 (3)	0.0202 (10)	
H7	0.743876	0.895536	0.486151	0.024*	
C8	1.1867 (6)	1.0247 (4)	0.3391 (3)	0.0236 (10)	
H8	1.212122	1.096675	0.329570	0.028*	
C9	0.3371 (7)	0.5597 (4)	0.1828 (4)	0.0262 (11)	
H9	0.395210	0.524102	0.239319	0.031*	
C10	0.4641 (6)	0.8072 (4)	0.5688 (3)	0.0228 (10)	
C11	1.2949 (6)	0.9415 (4)	0.3053 (3)	0.0228 (10)	
H11	1.403062	0.949758	0.269770	0.027*	
C12	0.6081 (6)	0.7719 (4)	0.4979 (3)	0.0177 (9)	
C13	1.0207 (6)	0.3657 (4)	0.2642 (3)	0.0233 (10)	
C14	0.5571 (7)	0.7789 (4)	−0.0537 (3)	0.0265 (11)	
H14	0.545588	0.783420	−0.117552	0.032*	
C15	0.6490 (6)	0.7205 (4)	0.1018 (3)	0.0200 (10)	
C16	0.5181 (6)	0.8177 (4)	0.0915 (3)	0.0217 (10)	
H16	0.477551	0.851307	0.139181	0.026*	
C17	0.6710 (6)	0.6982 (4)	0.0107 (3)	0.0237 (10)	
H17	0.747661	0.640105	−0.003396	0.028*	
C18	0.8253 (6)	0.5450 (4)	0.1969 (3)	0.0211 (10)	
H18	0.826535	0.520855	0.143234	0.025*	
C19	0.2517 (7)	0.6062 (4)	0.0283 (4)	0.0298 (12)	
H19	0.244149	0.606406	−0.034824	0.036*	
C20	0.3657 (7)	0.5287 (4)	0.0976 (4)	0.0291 (11)	
H20	0.444283	0.468764	0.088103	0.035*	
C21	0.2064 (6)	0.6533 (4)	0.1682 (4)	0.0274 (11)	
H21	0.162300	0.689881	0.213413	0.033*	
C22	0.1531 (7)	0.6822 (4)	0.0725 (4)	0.0302 (12)	
H22	0.067918	0.741102	0.044001	0.036*	
C23	0.4620 (7)	0.8529 (4)	−0.0039 (4)	0.0279 (11)	
H23	0.377786	0.913761	−0.029743	0.034*	
C24	1.0143 (9)	0.9500 (5)	0.1257 (4)	0.0403 (15)	
H24	1.073099	0.891183	0.104610	0.048*	
C25	0.8582 (8)	0.9484 (5)	0.1816 (4)	0.0435 (16)	
H25	0.792024	0.888831	0.204026	0.052*	
C26	0.8148 (8)	1.0523 (6)	0.1993 (4)	0.0488 (18)	
H26	0.716692	1.073614	0.236117	0.059*	
C27	1.0699 (9)	1.0535 (6)	0.1059 (4)	0.0450 (16)	
H27	1.171731	1.076386	0.068628	0.054*	
C28	0.9490 (10)	1.1174 (5)	0.1505 (4)	0.0504 (19)	
H28	0.955198	1.190510	0.148564	0.061*	
H6A	0.867 (6)	0.417 (3)	0.556 (4)	0.036 (16)*	
H5A	0.445 (4)	0.530 (5)	0.403 (5)	0.06 (2)*	
H5B	0.563 (7)	0.434 (2)	0.438 (3)	0.037 (16)*	
H6B	0.847 (7)	0.513 (5)	0.588 (4)	0.042 (17)*	

### Atomic displacement parameters (Å^2^)

**Table:**

	*U*^11^	*U*^22^	*U*^33^	*U*^12^	*U*^13^	*U*^23^
Fe1	0.0234 (4)	0.0162 (4)	0.0139 (4)	−0.0039 (3)	−0.0056 (3)	−0.0031 (3)
Co1	0.0160 (4)	0.0165 (4)	0.0152 (4)	−0.0017 (3)	−0.0038 (3)	−0.0047 (3)
Fe2	0.0224 (4)	0.0207 (4)	0.0158 (4)	−0.0050 (3)	−0.0057 (3)	−0.0033 (3)
F1	0.0244 (15)	0.0303 (16)	0.0402 (17)	0.0054 (12)	−0.0077 (13)	−0.0103 (13)
O4	0.0176 (16)	0.0186 (16)	0.0195 (16)	−0.0010 (13)	−0.0026 (12)	−0.0057 (13)
O3	0.0177 (15)	0.0132 (15)	0.0192 (16)	0.0002 (12)	−0.0047 (12)	−0.0043 (13)
O6	0.0207 (17)	0.0171 (16)	0.0180 (16)	0.0002 (13)	−0.0062 (13)	−0.0048 (13)
O2	0.0219 (16)	0.0174 (15)	0.0164 (16)	−0.0025 (13)	−0.0029 (13)	−0.0054 (13)
F3	0.0487 (19)	0.0396 (18)	0.0304 (16)	0.0089 (15)	−0.0063 (14)	−0.0211 (14)
O1	0.0194 (16)	0.0218 (16)	0.0174 (16)	−0.0016 (13)	−0.0033 (13)	−0.0065 (13)
O5	0.0191 (17)	0.0212 (17)	0.0193 (17)	−0.0050 (14)	−0.0031 (13)	−0.0050 (14)
F2	0.0389 (18)	0.0229 (16)	0.060 (2)	−0.0096 (13)	0.0134 (16)	−0.0146 (15)
F6	0.0221 (16)	0.088 (3)	0.0292 (17)	0.0146 (16)	−0.0079 (13)	−0.0228 (17)
F4	0.0391 (19)	0.068 (2)	0.0193 (16)	0.0164 (17)	−0.0001 (13)	−0.0002 (15)
F5	0.063 (2)	0.067 (3)	0.114 (4)	−0.036 (2)	0.053 (2)	−0.072 (3)
C1	0.021 (2)	0.017 (2)	0.009 (2)	0.0005 (18)	−0.0063 (17)	−0.0013 (17)
C2	0.019 (2)	0.020 (2)	0.020 (2)	−0.0068 (19)	0.0022 (18)	−0.0101 (19)
C3	0.014 (2)	0.022 (2)	0.015 (2)	−0.0014 (18)	−0.0052 (17)	−0.0035 (18)
C4	0.020 (2)	0.022 (2)	0.017 (2)	−0.0081 (19)	−0.0056 (18)	−0.0047 (19)
C5	0.027 (2)	0.020 (2)	0.016 (2)	0.0002 (19)	−0.0092 (19)	−0.0069 (19)
C6	0.021 (2)	0.022 (2)	0.017 (2)	0.0020 (19)	−0.0075 (18)	−0.0065 (19)
C7	0.023 (2)	0.019 (2)	0.020 (2)	−0.0018 (19)	−0.0012 (18)	−0.0093 (19)
C8	0.031 (3)	0.021 (2)	0.023 (2)	−0.010 (2)	−0.008 (2)	−0.006 (2)
C9	0.033 (3)	0.022 (2)	0.022 (2)	−0.012 (2)	−0.005 (2)	0.000 (2)
C10	0.021 (2)	0.024 (2)	0.025 (3)	0.000 (2)	−0.0023 (19)	−0.011 (2)
C11	0.019 (2)	0.032 (3)	0.019 (2)	−0.008 (2)	−0.0031 (18)	−0.006 (2)
C12	0.018 (2)	0.016 (2)	0.017 (2)	0.0064 (18)	−0.0060 (18)	−0.0063 (18)
C13	0.022 (2)	0.025 (2)	0.024 (2)	−0.003 (2)	0.0011 (19)	−0.009 (2)
C14	0.032 (3)	0.029 (3)	0.017 (2)	−0.010 (2)	−0.006 (2)	−0.001 (2)
C15	0.021 (2)	0.022 (2)	0.018 (2)	−0.0063 (19)	−0.0060 (18)	−0.0040 (19)
C16	0.027 (2)	0.016 (2)	0.022 (2)	−0.0074 (19)	−0.003 (2)	−0.0024 (19)
C17	0.022 (2)	0.031 (3)	0.019 (2)	−0.009 (2)	−0.0016 (19)	−0.007 (2)
C18	0.025 (2)	0.023 (2)	0.019 (2)	−0.0030 (19)	−0.0049 (19)	−0.010 (2)
C19	0.034 (3)	0.035 (3)	0.024 (3)	−0.019 (2)	−0.007 (2)	−0.007 (2)
C20	0.036 (3)	0.022 (3)	0.031 (3)	−0.011 (2)	−0.004 (2)	−0.007 (2)
C21	0.026 (3)	0.034 (3)	0.023 (2)	−0.013 (2)	0.002 (2)	−0.008 (2)
C22	0.028 (3)	0.035 (3)	0.027 (3)	−0.008 (2)	−0.008 (2)	−0.004 (2)
C23	0.030 (3)	0.021 (2)	0.027 (3)	−0.010 (2)	−0.012 (2)	0.007 (2)
C24	0.070 (4)	0.030 (3)	0.022 (3)	0.017 (3)	−0.026 (3)	−0.014 (2)
C25	0.051 (4)	0.045 (4)	0.033 (3)	−0.024 (3)	−0.028 (3)	0.008 (3)
C26	0.030 (3)	0.081 (5)	0.024 (3)	0.019 (3)	−0.014 (2)	−0.010 (3)
C27	0.053 (4)	0.058 (4)	0.019 (3)	−0.021 (3)	−0.009 (3)	0.003 (3)
C28	0.102 (6)	0.018 (3)	0.035 (3)	0.000 (3)	−0.047 (4)	−0.002 (2)

### Geometric parameters (Å, º)

**Table:**

Fe1—C3	2.019 (4)	C3—C5	1.428 (6)
Fe1—C5	2.015 (4)	C3—C6	1.446 (6)
Fe1—C6	2.046 (4)	C4—C15	1.475 (6)
Fe1—C8	2.056 (5)	C4—C18	1.431 (6)
Fe1—C11	2.069 (5)	C5—H5	0.9300
Fe1—C24	2.032 (5)	C5—C8	1.409 (7)
Fe1—C25	2.031 (5)	C6—H6	0.9300
Fe1—C26	2.018 (5)	C6—C11	1.424 (7)
Fe1—C27	2.032 (5)	C7—H7	0.9300
Fe1—C28	2.024 (5)	C7—C12	1.363 (6)
Co1—O4	2.042 (3)	C8—H8	0.9300
Co1—O3	2.082 (3)	C8—C11	1.418 (7)
Co1—O6	2.200 (3)	C9—H9	0.9300
Co1—O2	2.056 (3)	C9—C20	1.408 (7)
Co1—O1	2.031 (3)	C9—C21	1.404 (7)
Co1—O5	2.117 (3)	C10—C12	1.540 (6)
Fe2—C9	2.040 (5)	C11—H11	0.9300
Fe2—C14	2.056 (5)	C14—H14	0.9300
Fe2—C15	2.033 (4)	C14—C17	1.408 (7)
Fe2—C16	2.046 (4)	C14—C23	1.431 (7)
Fe2—C17	2.037 (5)	C15—C16	1.440 (7)
Fe2—C19	2.048 (5)	C15—C17	1.434 (6)
Fe2—C20	2.050 (5)	C16—H16	0.9300
Fe2—C21	2.052 (5)	C16—C23	1.415 (7)
Fe2—C22	2.059 (5)	C17—H17	0.9300
Fe2—C23	2.058 (5)	C18—H18	0.9300
F1—C13	1.334 (5)	C19—H19	0.9300
O4—C12	1.280 (5)	C19—C20	1.430 (7)
O3—C1	1.267 (5)	C19—C22	1.407 (8)
O6—H6A	0.88 (2)	C20—H20	0.9300
O6—H6B	0.89 (2)	C21—H21	0.9300
O2—C4	1.258 (5)	C21—C22	1.418 (7)
F3—C13	1.338 (5)	C22—H22	0.9300
O1—C2	1.283 (5)	C23—H23	0.9300
O5—H5A	0.89 (2)	C24—H24	0.9300
O5—H5B	0.88 (2)	C24—C25	1.369 (9)
F2—C13	1.324 (6)	C24—C27	1.381 (8)
F6—C10	1.310 (6)	C25—H25	0.9300
F4—C10	1.330 (6)	C25—C26	1.409 (9)
F5—C10	1.323 (6)	C26—H26	0.9300
C1—C3	1.459 (6)	C26—C28	1.408 (10)
C1—C7	1.442 (6)	C27—H27	0.9300
C2—C13	1.536 (6)	C27—C28	1.380 (9)
C2—C18	1.362 (6)	C28—H28	0.9300
			
C3—Fe1—C6	41.69 (17)	C8—C5—Fe1	71.3 (3)
C3—Fe1—C8	68.92 (18)	C8—C5—C3	108.7 (4)
C3—Fe1—C11	68.98 (17)	C8—C5—H5	125.6
C3—Fe1—C24	129.2 (2)	Fe1—C6—H6	126.5
C3—Fe1—C25	108.7 (2)	C3—C6—Fe1	68.2 (2)
C3—Fe1—C27	167.1 (2)	C3—C6—H6	126.2
C3—Fe1—C28	151.3 (3)	C11—C6—Fe1	70.6 (3)
C5—Fe1—C3	41.46 (18)	C11—C6—C3	107.5 (4)
C5—Fe1—C6	69.37 (18)	C11—C6—H6	126.2
C5—Fe1—C8	40.50 (19)	C1—C7—H7	118.3
C5—Fe1—C11	68.30 (19)	C12—C7—C1	123.4 (4)
C5—Fe1—C24	166.2 (2)	C12—C7—H7	118.3
C5—Fe1—C25	128.1 (2)	Fe1—C8—H8	127.2
C5—Fe1—C26	106.1 (2)	C5—C8—Fe1	68.2 (2)
C5—Fe1—C27	150.9 (2)	C5—C8—H8	125.8
C5—Fe1—C28	116.8 (2)	C5—C8—C11	108.4 (4)
C6—Fe1—C8	68.35 (19)	C11—C8—Fe1	70.4 (3)
C6—Fe1—C11	40.50 (18)	C11—C8—H8	125.8
C8—Fe1—C11	40.20 (19)	Fe2—C9—H9	125.2
C24—Fe1—C6	110.3 (2)	C20—C9—Fe2	70.3 (3)
C24—Fe1—C8	153.1 (2)	C20—C9—H9	125.7
C24—Fe1—C11	121.0 (2)	C21—C9—Fe2	70.4 (3)
C25—Fe1—C6	120.4 (2)	C21—C9—H9	125.7
C25—Fe1—C8	165.0 (3)	C21—C9—C20	108.6 (4)
C25—Fe1—C11	154.1 (2)	F6—C10—F4	106.0 (4)
C25—Fe1—C24	39.4 (3)	F6—C10—F5	107.2 (4)
C25—Fe1—C27	66.7 (2)	F6—C10—C12	112.6 (4)
C26—Fe1—C3	117.4 (2)	F4—C10—C12	110.2 (4)
C26—Fe1—C6	153.2 (3)	F5—C10—F4	105.5 (4)
C26—Fe1—C8	126.1 (2)	F5—C10—C12	114.9 (4)
C26—Fe1—C11	164.1 (3)	Fe1—C11—H11	127.5
C26—Fe1—C24	67.6 (2)	C6—C11—Fe1	68.9 (2)
C26—Fe1—C25	40.7 (3)	C6—C11—H11	125.8
C26—Fe1—C27	67.6 (3)	C8—C11—Fe1	69.4 (3)
C26—Fe1—C28	40.8 (3)	C8—C11—C6	108.3 (4)
C27—Fe1—C6	129.1 (2)	C8—C11—H11	125.8
C27—Fe1—C8	118.8 (2)	O4—C12—C7	130.3 (4)
C27—Fe1—C11	109.6 (2)	O4—C12—C10	112.4 (4)
C27—Fe1—C24	39.7 (2)	C7—C12—C10	117.3 (4)
C28—Fe1—C6	165.2 (3)	F1—C13—F3	106.3 (4)
C28—Fe1—C8	106.9 (2)	F1—C13—C2	111.4 (4)
C28—Fe1—C11	127.1 (2)	F3—C13—C2	113.7 (4)
C28—Fe1—C24	67.2 (2)	F2—C13—F1	106.9 (4)
C28—Fe1—C25	67.7 (2)	F2—C13—F3	107.2 (4)
C28—Fe1—C27	39.8 (3)	F2—C13—C2	110.9 (4)
O4—Co1—O3	88.20 (12)	Fe2—C14—H14	126.8
O4—Co1—O6	84.49 (12)	C17—C14—Fe2	69.1 (3)
O4—Co1—O2	99.68 (12)	C17—C14—H14	125.9
O4—Co1—O5	91.75 (12)	C17—C14—C23	108.1 (4)
O3—Co1—O6	93.19 (11)	C23—C14—Fe2	69.7 (3)
O3—Co1—O5	177.31 (12)	C23—C14—H14	125.9
O2—Co1—O3	92.11 (12)	C4—C15—Fe2	120.1 (3)
O2—Co1—O6	173.36 (12)	C16—C15—Fe2	69.8 (3)
O2—Co1—O5	85.25 (12)	C16—C15—C4	124.5 (4)
O1—Co1—O4	170.17 (12)	C17—C15—Fe2	69.5 (3)
O1—Co1—O3	93.61 (12)	C17—C15—C4	128.0 (4)
O1—Co1—O6	85.76 (12)	C17—C15—C16	107.1 (4)
O1—Co1—O2	89.91 (12)	Fe2—C16—H16	126.4
O1—Co1—O5	86.89 (13)	C15—C16—Fe2	68.8 (2)
O5—Co1—O6	89.48 (12)	C15—C16—H16	126.0
C9—Fe2—C14	161.3 (2)	C23—C16—Fe2	70.3 (3)
C9—Fe2—C16	121.12 (19)	C23—C16—C15	108.0 (4)
C9—Fe2—C19	68.09 (19)	C23—C16—H16	126.0
C9—Fe2—C20	40.3 (2)	Fe2—C17—H17	126.0
C9—Fe2—C21	40.1 (2)	C14—C17—Fe2	70.6 (3)
C9—Fe2—C22	67.8 (2)	C14—C17—C15	108.6 (4)
C9—Fe2—C23	156.4 (2)	C14—C17—H17	125.7
C14—Fe2—C22	122.4 (2)	C15—C17—Fe2	69.2 (3)
C14—Fe2—C23	40.7 (2)	C15—C17—H17	125.7
C15—Fe2—C9	106.95 (19)	C2—C18—C4	124.5 (4)
C15—Fe2—C14	68.69 (18)	C2—C18—H18	117.7
C15—Fe2—C16	41.35 (18)	C4—C18—H18	117.7
C15—Fe2—C17	41.26 (18)	Fe2—C19—H19	125.5
C15—Fe2—C19	157.4 (2)	C20—C19—Fe2	69.7 (3)
C15—Fe2—C20	121.1 (2)	C20—C19—H19	126.1
C15—Fe2—C21	123.60 (19)	C22—C19—Fe2	70.4 (3)
C15—Fe2—C22	160.5 (2)	C22—C19—H19	126.1
C15—Fe2—C23	68.74 (19)	C22—C19—C20	107.8 (4)
C16—Fe2—C14	68.40 (19)	Fe2—C20—H20	126.4
C16—Fe2—C19	160.3 (2)	C9—C20—Fe2	69.5 (3)
C16—Fe2—C20	156.71 (19)	C9—C20—C19	107.5 (5)
C16—Fe2—C21	107.2 (2)	C9—C20—H20	126.3
C16—Fe2—C22	123.9 (2)	C19—C20—Fe2	69.5 (3)
C16—Fe2—C23	40.34 (18)	C19—C20—H20	126.3
C17—Fe2—C9	124.7 (2)	Fe2—C21—H21	126.1
C17—Fe2—C14	40.23 (19)	C9—C21—Fe2	69.5 (3)
C17—Fe2—C16	69.0 (2)	C9—C21—H21	125.9
C17—Fe2—C19	122.1 (2)	C9—C21—C22	108.1 (5)
C17—Fe2—C20	108.1 (2)	C22—C21—Fe2	70.1 (3)
C17—Fe2—C21	160.95 (19)	C22—C21—H21	125.9
C17—Fe2—C22	157.2 (2)	Fe2—C22—H22	126.4
C17—Fe2—C23	68.3 (2)	C19—C22—Fe2	69.5 (3)
C19—Fe2—C14	108.5 (2)	C19—C22—C21	107.9 (5)
C19—Fe2—C20	40.9 (2)	C19—C22—H22	126.0
C19—Fe2—C21	67.7 (2)	C21—C22—Fe2	69.6 (3)
C19—Fe2—C22	40.1 (2)	C21—C22—H22	126.0
C19—Fe2—C23	124.7 (2)	Fe2—C23—H23	126.7
C20—Fe2—C14	125.2 (2)	C14—C23—Fe2	69.6 (3)
C20—Fe2—C21	67.6 (2)	C14—C23—H23	125.9
C20—Fe2—C22	67.9 (2)	C16—C23—Fe2	69.4 (3)
C20—Fe2—C23	161.9 (2)	C16—C23—C14	108.2 (4)
C21—Fe2—C14	157.4 (2)	C16—C23—H23	125.9
C21—Fe2—C22	40.35 (19)	Fe1—C24—H24	125.4
C21—Fe2—C23	121.6 (2)	C25—C24—Fe1	70.3 (3)
C23—Fe2—C22	108.1 (2)	C25—C24—H24	125.7
C12—O4—Co1	124.6 (3)	C25—C24—C27	108.5 (5)
C1—O3—Co1	127.0 (3)	C27—C24—Fe1	70.1 (3)
Co1—O6—H6A	118 (4)	C27—C24—H24	125.7
Co1—O6—H6B	114 (4)	Fe1—C25—H25	126.3
H6A—O6—H6B	108 (5)	C24—C25—Fe1	70.4 (3)
C4—O2—Co1	123.8 (3)	C24—C25—H25	125.8
C2—O1—Co1	120.9 (3)	C24—C25—C26	108.4 (5)
Co1—O5—H5A	119 (4)	C26—C25—Fe1	69.1 (3)
Co1—O5—H5B	116 (4)	C26—C25—H25	125.8
H5A—O5—H5B	103 (6)	Fe1—C26—H26	124.9
O3—C1—C3	118.7 (4)	C25—C26—Fe1	70.2 (3)
O3—C1—C7	123.3 (4)	C25—C26—H26	126.7
C7—C1—C3	118.0 (4)	C28—C26—Fe1	69.8 (3)
O1—C2—C13	112.7 (4)	C28—C26—C25	106.6 (6)
O1—C2—C18	129.0 (4)	C28—C26—H26	126.7
C18—C2—C13	118.2 (4)	Fe1—C27—H27	126.0
C1—C3—Fe1	118.9 (3)	C24—C27—Fe1	70.1 (3)
C5—C3—Fe1	69.1 (2)	C24—C27—H27	125.7
C5—C3—C1	128.0 (4)	C28—C27—Fe1	69.8 (3)
C5—C3—C6	107.0 (4)	C28—C27—C24	108.7 (6)
C6—C3—Fe1	70.1 (2)	C28—C27—H27	125.7
C6—C3—C1	124.4 (4)	Fe1—C28—H28	125.7
O2—C4—C15	117.4 (4)	C26—C28—Fe1	69.4 (3)
O2—C4—C18	125.1 (4)	C26—C28—H28	126.1
C18—C4—C15	117.5 (4)	C27—C28—Fe1	70.4 (3)
Fe1—C5—H5	125.2	C27—C28—C26	107.8 (5)
C3—C5—Fe1	69.4 (2)	C27—C28—H28	126.1
C3—C5—H5	125.6		

### Hydrogen-bond geometry (Å, º)

**Table:**

*D*—H···*A*	*D*—H	H···*A*	*D*···*A*	*D*—H···*A*
O5—H5*A*···O6^i^	0.89 (4)	1.97 (4)	2.797 (5)	154 (6)
O5—H5*B*···O4^i^	0.88 (4)	2.01 (4)	2.781 (4)	146 (5)
O6—H6*A*···O3^ii^	0.88 (4)	1.91 (5)	2.739 (5)	156 (4)
O6—H6*B*···O1^ii^	0.89 (6)	1.99 (6)	2.741 (5)	141 (5)
C25—H25···O2	0.93	2.57	3.467 (7)	163
C28—H28···F3^iii^	0.93	2.55	3.449 (8)	163

Symmetry codes: (i) −*x*+1, −*y*+1, −*z*+1; (ii) −*x*+2, −*y*+1, −*z*+1; (iii) *x*, *y*+1, *z*.
